# Comparison of Long-Term Oral Bacterial Flora Before and After Orthognathic Surgery in Surgical Orthodontic Treatment

**DOI:** 10.3390/dj13100458

**Published:** 2025-10-08

**Authors:** Rumi Matsumoto, Masahiro Takahashi, Kazuyoshi Hosomichi, Satoko Okuwaki, So Koizumi, Yu Hikita, Reina Hatanaka, Tetsutaro Yamaguchi

**Affiliations:** 1 Department of Orthodontics, School of Dentistry, Kanagawa Dental University, 82 Inaoka-cho, Yokosuka 238-8580, Japan; takahashi.masahiro@kdu.ac.jp (M.T.); okuwaki@kdu.ac.jp (S.O.); koizumi@kdu.ac.jp (S.K.); hikita-yu@dent.showa-u.ac.jp (Y.H.); hatanaka@kdu.ac.jp (R.H.); t.yamaguchi@kdu.ac.jp (T.Y.); 2 Laboratory of Computational Genomics, School of Life Science, Tokyo University of Pharmacy and Life Sciences, 1432-1 Horinouchi, Hachioji 192-0392, Japan; khosomic@toyaku.ac.jp

**Keywords:** oral microbiota, multi-bracket appliance, orthognathic surgery, surgical orthodontics, 16S rRNA gene sequencing

## Abstract

**Background/Objectives:** Multi-bracket appliances are essential in surgical orthodontic treatment, and perioperative oral management during orthognathic surgery is critical. Thorough plaque control, appropriate use of antibiotics, and shortening of operative time have been reported to be effective in preventing postoperative infections and ensuring surgical success. As highly invasive orthognathic surgery involving osteotomy may influence the postoperative oral microbiota, this study aimed to investigate the characteristics of and clarify the changes occurring in the salivary oral microbiota after orthognathic surgery. **Methods:** The study included 14 patients (Group S; mean age 29.3 ± 9.8 years) who underwent surgical orthodontic treatment and 15 control patients (Group C; mean age 27.1 ± 8.7 years) who received orthodontic treatment alone. Salivary samples were analyzed via 16S rRNA gene sequencing, and the relative abundances of bacteria were evaluated using the Linear Discriminant Analysis Effect Size. **Results:** The prevalence of *Neisseria*, which is associated with early biofilm formation, decreased over time in both groups. In contrast, *Streptococcus* exhibited an increase in prevalence. In Group S, members of *Pseudomonas*, the family Saccharimonadaceae, and the order Rhizobiales showed increases at 5–8 months post-surgery. **Conclusions:** Surgical orthodontic treatment may influence the oral microbiota and promote colonization by opportunistic pathogens. Instructions regarding oral hygiene and appropriately timed professional cleaning interventions are critical in preventing such colonization. Longitudinal monitoring of the microbiota using metagenomic analysis may be useful for future perioperative management and guidance of oral hygiene.

## 1. Introduction

Jaw deformities are characterized by skeletal disharmony due to abnormalities in the morphology, size, or position of the maxilla and mandible. These deformities are often associated with malocclusion, which can be severe, and aesthetic facial disharmony [1]. Surgical orthodontic treatment combined with orthognathic surgery is generally performed to correct intermaxillary discrepancies resulting from maxillomandibular deformities or asymmetry in the permanent dentition. Although aesthetic concerns are common, patients often present with multiple functional problems, including masticatory dysfunction, speech disorders, and obstructive sleep apnea syndrome [2].

Multi-bracket appliances (MBAs) are an essential part of surgical orthodontic treatment. However, as they are fixed appliances, patients treated with MBAs have been reported to present a significantly increased quantity and altered quality of supragingival and subgingival plaques when compared with those without such appliances, thereby increasing the risk of caries and periodontal disease [3,4,5]. Perioperative oral management is therefore crucial, and measures such as thorough plaque control, appropriate antibiotic administration, and shortening of operative time have been reported to effectively prevent postoperative infections and ensure surgical success [6]. In contrast, prolonged surgery and bimaxillary osteotomy are known risk factors for surgical site infection (SSI), and highly invasive orthognathic surgery involving osteotomy may thus affect the postoperative oral microbiota.

During orthognathic surgery, oral bacteria can migrate between the oral cavity and internal tissues until wound healing is complete. Opportunistic infections can occur as a result of disruption of immune balance, and these biological changes have been linked to growth, hormonal fluctuations, reduced salivary flow, gingivitis, periodontitis, diet, smoking, and poor oral hygiene [4,7]. The mandible, due to its poorer vascular supply than the maxilla, as well as the tendency of saliva and food debris to stagnate in its vestibular incision area, is considered to be more susceptible to SSI following bilateral sagittal split osteotomy or intraoral vertical ramus osteotomy, when compared with Le Fort osteotomy of the maxilla [8].

Although previous studies have investigated changes in the salivary microbiota [3,4,5,9,10,11,12,13,14,15] or the short-term effects of orthognathic surgery on periodontal tissues and the oral microbiota [16], few have examined the long-term changes occurring after surgical orthodontic treatment. Moreover, the influences of orthodontic appliances and perioperative antibiotics have already been described, but the combined impact of surgical intervention and long-term orthodontic treatment on the oral microbiota is still poorly understood. Therefore, the long-term microbial shifts and characteristics were investigated in this study in patients undergoing surgical orthodontic treatment compared with orthodontic treatment alone.

## 2. Materials and Methods

### 2.1. Ethical Statement

This study was conducted in accordance with the guidelines of the Declaration of Helsinki and followed the Strengthening the Reporting of Observational Studies in Epidemiology (STROBE) checklist. Ethical approval was obtained from the Research Ethics Committee of Kanagawa Dental University (Approval No. 1033). Written informed consent was obtained from all participants.

### 2.2. Participants

The participants were patients who received treatment at the Department of Orthodontics, Kanagawa Dental University Hospital, including those who underwent surgical orthodontic treatment (Group S) and those who received orthodontic treatment alone (Group C). Group S consisted of 14 patients (mean age 29.3 ± 9.8 years; 5 men and 9 women) treated with conventional MBAs. Group C consisted of 15 patients (mean age 27.1 ± 8.7 years; 4 men and 11 women) also treated with conventional MBAs, who were undergoing canine or incisor retraction. The participant data are detailed in Table 1.

Exclusion criteria included the presence of systemic disease, smoking, use of antibiotics or hormonal agents within 3 months prior to sampling, and difficulty in communication. Patients in Group S received intravenous amoxicillin 1.0 g during surgery, followed by a postoperative intravenous administration of amoxicillin for an average of 4.4 days (range: 4–9 days) at a mean daily dose of 1.8 ± 0.5 g.

Oral hygiene instructions were provided as appropriate, according to the condition of each patient, based on the discretion of the attending clinician after the initiation of active treatment with MBAs. The investigators did not specify any standardized method for brushing technique or toothbrush type (manual or electric). They determined the sample size of this study by referring to relevant previous studies: since similar research on microbiome changes after orthodontic treatment used comparable numbers of participants to ensure consistency and comparability, they regarded a sample of approximately 29 participants as sufficient to maintain validity [3,17].

### 2.3. Saliva Sampling

**Figure 1:**
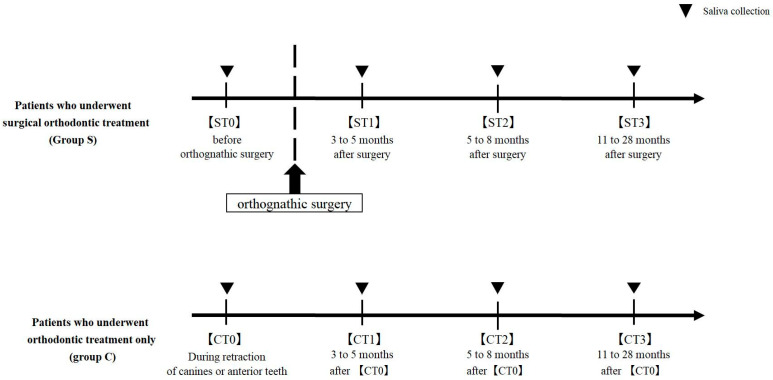
Flowchart of saliva collection in patients who underwent surgical orthodontic treatment (Group S) and those who received orthodontic treatment only (Group C). Group S: ST0 (before orthognathic surgery), ST1 (3–5 months after surgery), ST2 (5–8 months after surgery), and ST3 (11–28 months after surgery). Group C: CT0 (during retraction of canines or anterior teeth) and CT1–CT3 (3–28 months after CT0). Black triangles indicate saliva collection points.

To evaluate longitudinal changes in the oral microbiota, saliva samples were collected from all participants at four time points using the Oragene^®^ DISCOVER kit (DNA Genotek, Ottawa, ON, Canada). The sampling frequency was determined based on previous studies [13], and each sample was collected within an average of 20 min. In Group S, saliva was collected at the following time points: within 2 months before orthognathic surgery (ST0), at 3–5 months post-surgery (ST1), at 5–8 months post-surgery (ST2), and at 11 months to 2 years 4 months post-surgery (ST3). We selected control group cases with canine or incisor traction because the duration required for treatment completion was nearly the same as that of the surgical orthodontic group. In Group C, saliva was collected at the start of canine or incisor retraction (CT0), and at time points corresponding to those for Group S: CT1 (3–5 months after CT0), CT2 (5–8 months after CT0), and CT3 (11 months to 2 years 4 months after CT0). The sample collection flow chart is shown in Figure 1.

### 2.4. Microbial DNA Extraction

#### 2.4.1. DNA Extraction from Saliva Samples

Genomic DNA was extracted from saliva samples according to the manufacturer’s protocol. DNA concentrations were measured using the Qubit^®^ dsDNA High Sensitivity Assay Kit (Thermo Fisher Scientific, Waltham, MA, USA), and the extracted DNA was used for subsequent 16S rRNA amplicon analysis.

#### 2.4.2. Amplicon Generation and Library Preparation

Sequencing libraries were constructed using the MetaVX Library Preparation Kit (GENEWIZ, Suzhou, China). Amplicons covering the V3 and V4 hypervariable regions of the bacterial 16S rRNA gene were generated using 20–50 ng of DNA. The forward primer sequence was ACTCCTACGGGAGGAGGCAGCAG, and the reverse primer sequence was GGACTACHVGGGTWTCTAAT. The DNA concentrations were measured using a microplate reader (Tecan Infinite^®^ 200 Pro, Tecan Group Ltd., Männedorf, Switzerland).

Polymerase chain reaction (PCR) amplification was carried out in two steps using TransStart Taq DNA polymerase.

In the first round, a 25 μL reaction mixture was prepared, consisting of 18 μL genomic DNA template, 2.5 μL 10× TransStart Buffer, 2 μL dNTPs (2.5 mM each), 2 μL each of forward and reverse primers, and 0.5 μL TransStart Taq DNA polymerase (2.5 U/μL). Cycling was conducted under the following conditions: initial denaturation at 94 °C for 3 min; 14–16 cycles of 94 °C for 30 s, 53 °C for 30 s, and 72 °C for 30 s; followed by a final extension at 72 °C for 5 min.

In the second round, 25 μL of the first-round product was used as a template in a 100 μL reaction containing 2.5 μL 10× TransStart Buffer, 2 μL dNTPs (2.5 mM each), 3 μL each of index primers N and S, 4 μL of 1× cocktail, and 0.5 μL TransStart Taq DNA polymerase (2.5 U/μL). The thermal cycling program consisted of an initial denaturation at 94 °C for 3 min; 10–12 cycles at 94 °C for 10 s, 60 °C for 30 s, and 72 °C for 15 s; and a final extension at 72 °C for 5 min.

The PCR products were subsequently purified and subjected to quality control. The fragment size (approximately 600 bp) was verified using 1.5% agarose gel electrophoresis, and DNA concentrations were re-assessed with a microplate reader (Tecan Infinite^®^ 200 Pro, Tecan Group Ltd., Männedorf, Switzerland).

### 2.5. Sequencing

Sequencing was performed on the Illumina NovaSeq platform (Illumina, San Diego, CA, USA) using 250 or 300 bp paired-end reads. Cluster generation and sequencing were carried out automatically, according to the manufacturer’s protocol.

### 2.6. Bioinformatics Analysis

Raw sequencing data were processed using the DADA2 pipeline for quality filtering, noise removal, and chimera elimination to obtain amplicon sequence variants (ASVs). Taxonomic assignment of representative ASV sequences was performed using the SILVA 138 database and the Bayesian algorithm of the RDP classifier.

### 2.7. Statistical Analysis

Statistical analysis and data visualization were performed using the R software (version 3.3.1), and the Linear Discriminant Analysis Effect Size (LEfSe) was used to identify differentially abundant bacterial taxa between groups. Non-parametric tests (Kruskal–Wallis test and Wilcoxon rank-sum test) were performed based on the relative abundance of each taxon, followed by linear discriminant analysis (LDA) to evaluate effect sizes.

To extract characteristic microbial taxa at different taxonomic levels, an LDA score of ≥2.0 was considered statistically significant. To account for multiple testing, *p*-values were adjusted using the false discovery rate (FDR), and taxa with q < 0.05 were considered statistically significant. The analyses were conducted using the Galaxy-based LEfSe pipeline (http://galaxy.biobakery.org/, accessed on 26 January 2025) [18]. In addition, we calculated mean relative abundances for taxa that showed significant differences by dividing the number of reads assigned to each taxon by the total reads per sample and averaging these values across samples within each group. We reported these values together with LDA scores and adjusted *p*-values (q-values) to highlight the biological relevance of the results. We also conducted post hoc power analyses for taxa showing significant or near-significant differences. We estimated effect sizes from relative abundance data and calculated statistical power using G*Power (version 3.1.9.7). Finally, we reported both *p*-values and q-values together with effect sizes and post hoc power to clarify whether the study had sufficient power to detect the observed differences.

## 3. Results

### 3.1. Taxonomic Composition of the Oral Microbiota

**Figure 2:**
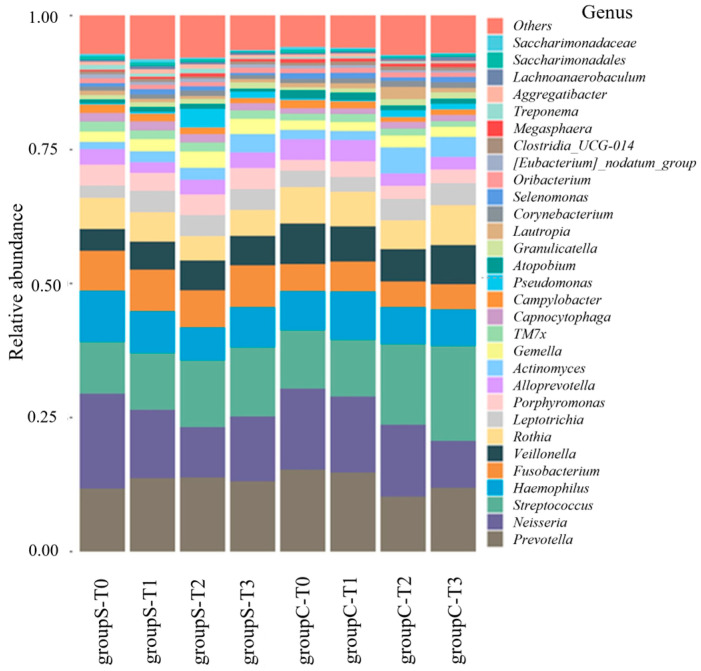
Relative abundance of bacterial genera in saliva samples from patients undergoing surgical orthodontic treatment (Group S) and orthodontic treatment only (Group C) at four time points (T0–T3). The *x*-axis indicates the group, and the *y*-axis displays the relative abundances of distinct bacterial taxa classified at the genus or species level.

In the present study, the salivary microbiota was classified into 13 phyla, 17 classes, 36 orders, 57 families, 96 genera, and 189 species. Classification from phylum to species level was performed using 16S rRNA amplicon analysis targeting the V3–V4 region. The classification results were highly consistent at the genus level, and considering the limitations of V3–V4 region-based classification, analyses were primarily conducted at the genus level. Comparison of the relative abundances of bacterial genera showed an increase in *Pseudomonas* at ST2 in Group S; in particular, although the relative abundance of Pseudomonas was generally low compared with dominant oral taxa (e.g., *Prevotella*, *Neisseria*, *Streptococcus*), it showed a statistically significant increase at ST2, with a mean of 0.38% (±0.43%) and a maximum of 0.67%. *Neisseria* showed a decreasing trend over time in both groups, whereas *Streptococcus* showed an increasing trend over time (Figure 2).

**Figure 3:**
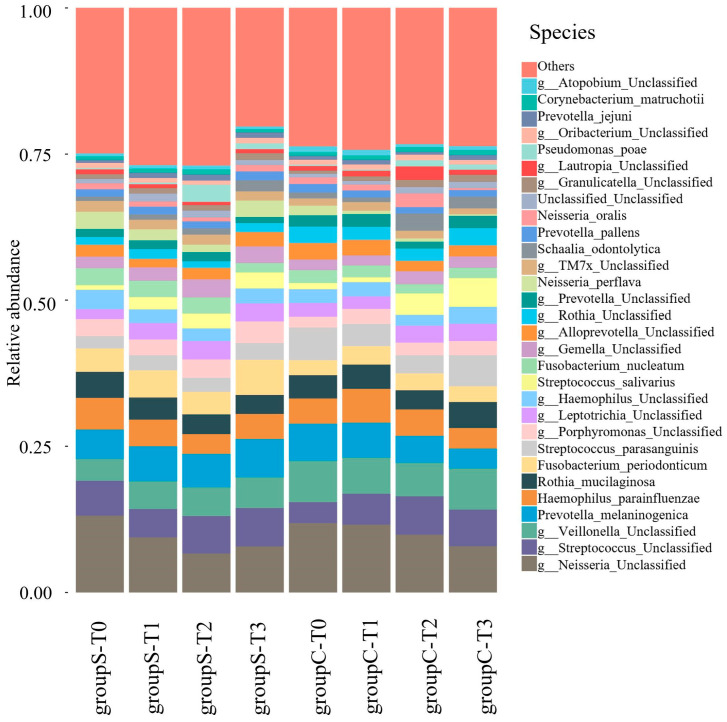
Relative abundance of bacterial species in saliva samples from patients undergoing surgical orthodontic treatment (Group S) or orthodontic treatment only (Group C) at four time points (T0–T3).

Comparison of the relative abundances at the species level showed a marked increase in *Pseudomonas* at ST2. An unidentified species derived from *Neisseria* showed a decreasing trend over time in both Groups S and C, whereas *Streptococcus* tended to increase over time (Figure 3).

### 3.2. LEfSe

#### 3.2.1. Comparison of Salivary Bacterial Composition at Each Sampling Point in Group S

**Figure 4:**
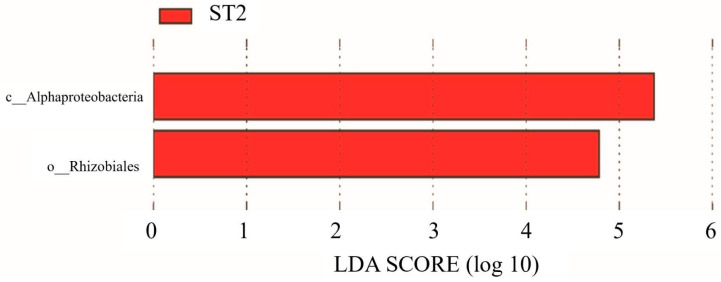
LDA results for ST0 vs. ST2 comparison in Group S (before surgery vs. 6 months after surgery) (*p* < 0.05).

No significant differences were detected between ST0 and ST1 regarding the salivary bacterial composition. An increase in Rhizobiales (order) (LDA score = 5.11, *p* = 0.035, q = 0.035, mean relative abundance = 4.78) was observed between ST0 and ST2 (Figure 4).

**Figure 5:**
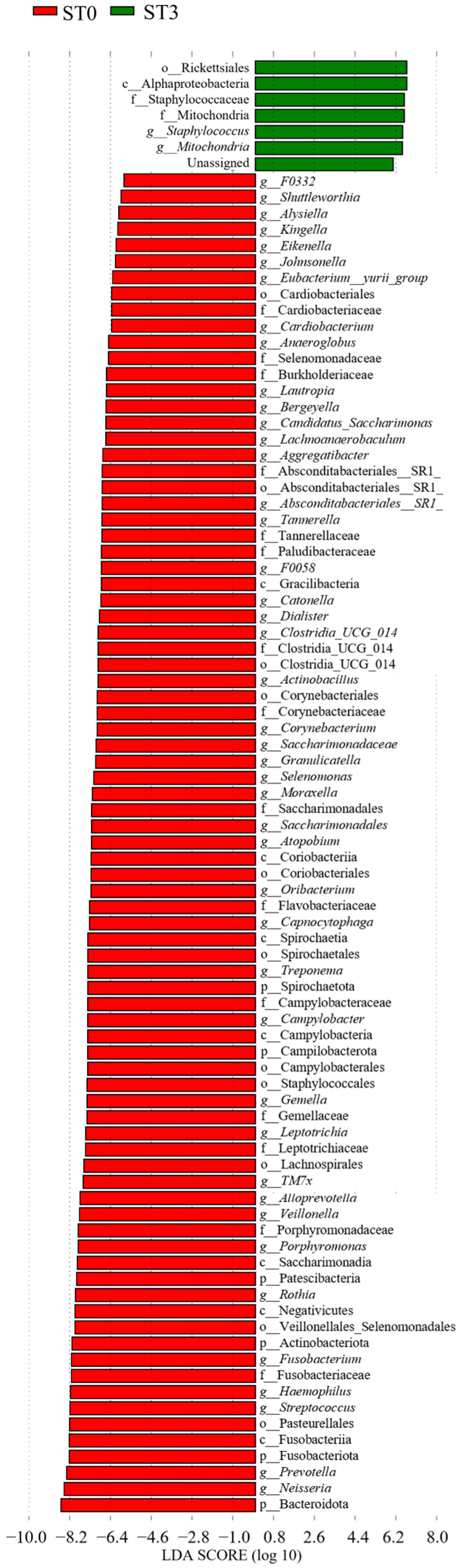
LDA results for ST0 vs. ST3 comparison in Group S (preoperative vs. 11 months to 2 years and 4 months postoperative) (*p* < 0.05).

At ST0, taxa comprising Gram-negative anaerobic bacteria and oral commensals, such as *Prevotella* (LDA score = 8.69, *p* = 0.021, q = 0.027, mean relative abundance = 8.33), *Neisseria* (LDA score = 8.75, *p* = 0.001, q = 0.011, mean relative abundance = 8.44), *Veillonella* (LDA score = 8.14, *p* = 0.024, q = 0.030, mean relative abundance = 7.76), *Campylobacter* (LDA score = 7.73, *p* = 0.002, q = 0.011, mean relative abundance = 7.40), and *Treponema* (LDA score = 7.66, *p* = 0.001, q = 0.011, mean relative abundance = 7.39), were detected with significantly greater abundances. In contrast, at ST3, *Staphylococcus* (genus) (LDA score = 4.54, *p* = 0.016, q = 0.022, mean relative abundance = 6.50) and Rickettsiales (order) (LDA score = 4.27, *p* = 0.040, q = 0.040, mean relative abundance = 6.68) were predominant (Figure 5).

**Figure 6:**
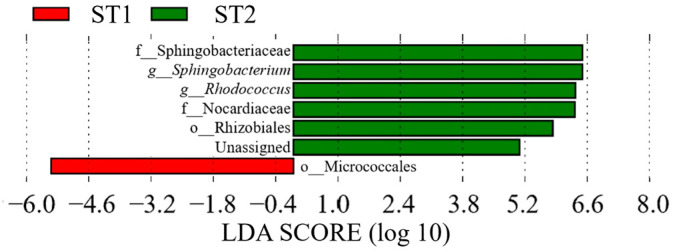
LDA results for ST1 vs. ST2 comparison in Group S (3–5 months vs. 5–8 months after surgery) (*p* < 0.05).

Comparing ST1 and ST2, taxa such as Sphingobacteriaceae (family) (LDA score = 6.88, *p* = 0.022, q = 0.035, mean relative abundance = 6.49), *Sphingobacterium* (genus) (LDA score = 6.88, *p* = 0.022, q = 0.035, mean relative abundance = 6.49), *Rhodococcus* (genus) (LDA score = 6.66, *p* = 0.035, q = 0.035, mean relative abundance = 6.33), Nocardiaceae (family) (*p* = 0.035), and Rhizobiales (order) (LDA score =5.11, *p* = 0.035, q = 0.035, mean relative abundance = 5.83) were detected with significantly greater abundance at ST2. In contrast, only Micrococcales (order) (LDA score = 5.83, *p* = 0.035, q = 0.035, mean relative abundance = 5.44) was detected in significantly greater abundance at ST1 (Figure 6).

**Figure 7:**
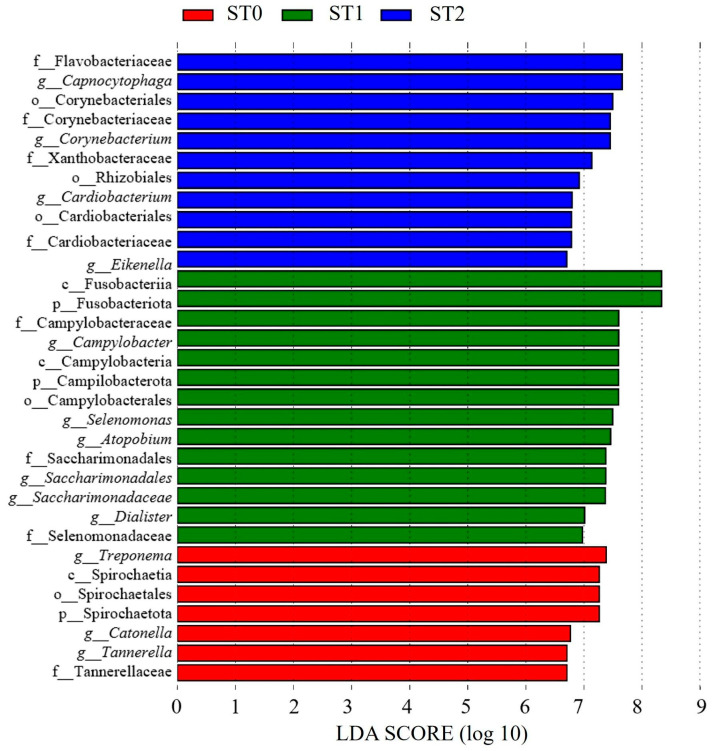
Bar chart showing the LDA scores (log 10) for Group S regarding differentially abundant microbial taxa across saliva samples obtained at ST0 (preoperative), ST1 (3–5 months postoperative), and ST2 (5–8 months postoperative). Colors indicate the time point at which each taxon was most significantly enriched (*p* < 0.05).

At ST0, periodontal disease-associated bacteria, such as *Treponema* (genus) (LDA score = 7.66, *p* = 0.007, q = 0.033, mean relative abundance = 7.38) and Spirochaetales (order) (LDA score = 7.66, *p* = 0.007, q = 0.033, mean relative abundance = 7.26), were predominant. Meanwhile, at ST1, there was a shift toward bacteria associated with inflammation and periodontal disease, such as Fusobacteriia (class) (LDA score = 8.62, *p* = 0.046, q = 0.047, mean relative abundance = 8.34) and *Campylobacter* (genus) (LDA score = 7.87, *p* = 0.017, q = 0.038, mean relative abundance = 7.59). In addition, bacteria commonly recognized as oral commensals, including *Corynebacterium* (genus) (LDA score = 7.77, *p* = 0.042, q = 0.046, mean relative abundance = 7.45), *Capnocytophaga* (genus) (LDA score = 7.89, *p* = 0.040, q = 0.046, mean relative abundance = 7.66), and *Eikenella* (genus) (LDA score = 6.94, *p* = 0.026, q = 0.044, mean relative abundance = 6.70), became more abundant at ST2 (Figure 7).

#### 3.2.2. Comparison of Salivary Bacterial Composition at Each Sampling Point Between Group S and Group C

**Figure 8:**
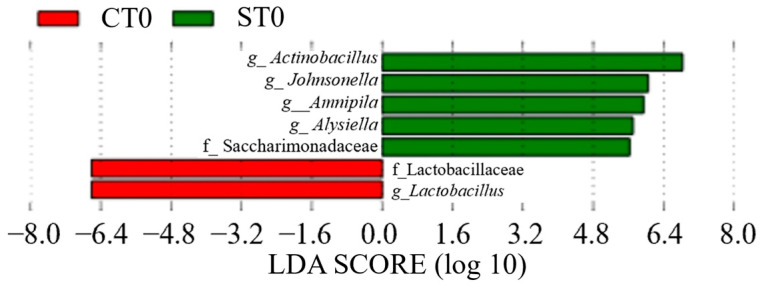
Comparison of preoperative samples between patients who underwent surgical orthodontic treatment (ST0) and those who received orthodontic treatment only (CT0) (*p* < 0.05). LDA, linear discriminant analysis.

**Figure 9:**
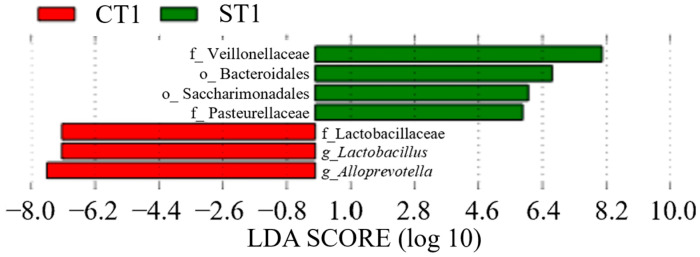
Comparison of post-treatment samples collected at 3–5 months after intervention between patients who underwent surgical orthodontic treatment (ST1) and those who received orthodontic treatment only (CT1) (*p* < 0.05). LDA, linear discriminant analysis.

**Figure 10:**
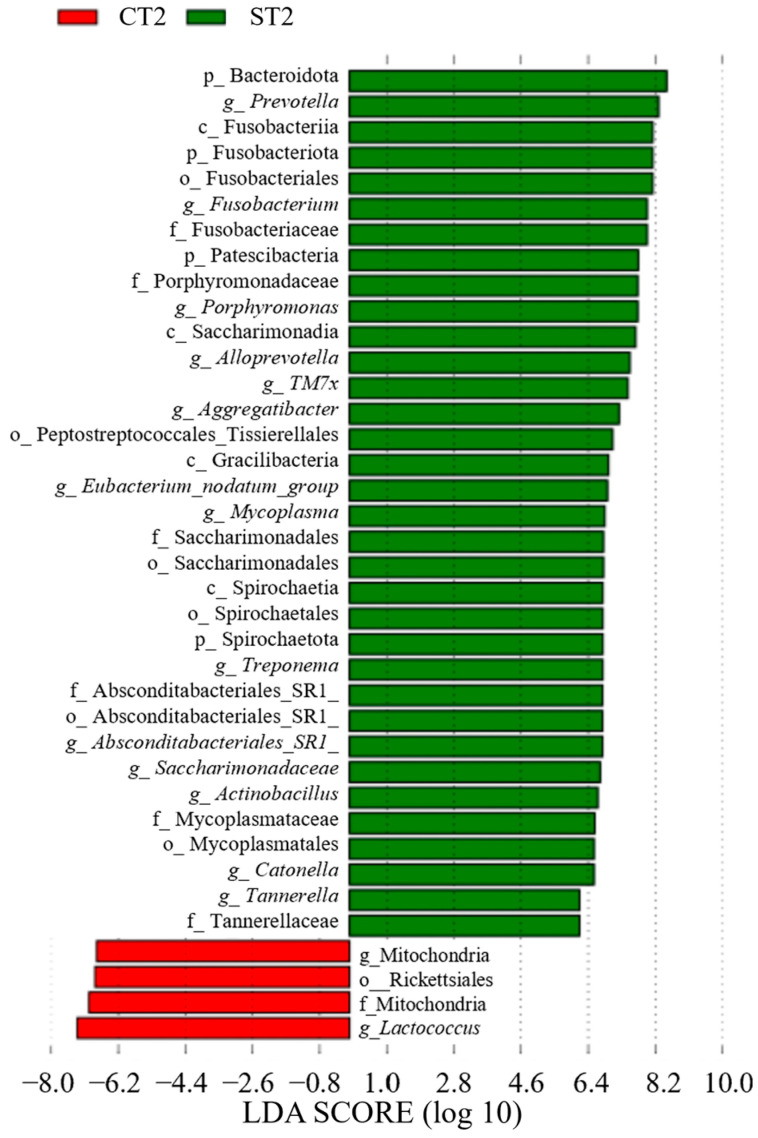
Comparison of post-treatment samples collected at 5–8 months after intervention between patients who underwent surgical orthodontic treatment (ST2) and those who received orthodontic treatment only (CT2) (*p* < 0.05). LDA, linear discriminant analysis.

**Figure 11:**
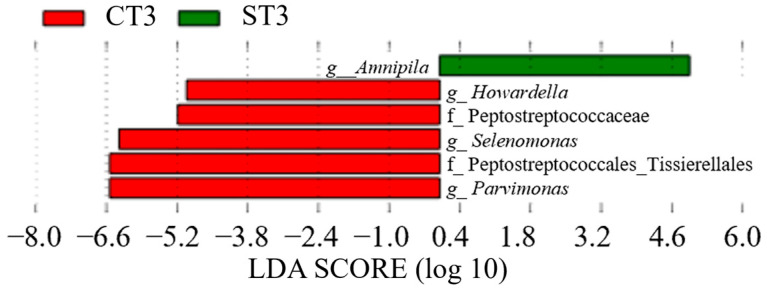
Comparison of post-treatment samples collected between 11 months and 2 years and 4 months after intervention between patients who underwent surgical orthodontic treatment (ST3) and those who received orthodontic treatment only (CT3) (*p* < 0.05). LDA, linear discriminant analysis.

In Group C, *Lactobacillus* (genus) was detected between CT0 (LDA score = 6.70, *p* = 0.035, q = 0.035, mean relative abundance = 6.60) and CT1 (LDA score = 6.09, *p* = 0.023, q = 0.050, mean relative abundance = 7.13) (Figure 8 and Figure 9). In contrast, in Group S, Bacteroidales (order) showed a significant increase at ST1 (LDA score = 5.02, *p* = 0.036, q = 0.050, mean relative abundance = 6.68), whereas *Bacteroidota* (phylum) showed a significant increase at ST2 (LDA score = 9.00, *p* = 0.032, q = 0.044, mean relative abundance = 8.50) (Figure 9 and Figure 10). A comparison between CT2 and ST2 revealed a significant increase in bacterial abundance in Group S (Figure 10) (*p* < 0.05). Furthermore, the presence of *Amnipila* (genus) appeared to be characteristic of ST3, as it was not detected at CT3 (LDA score = 5.35, *p* = 0.019, q = 0.042, mean relative abundance = 4.94) (Figure 11).

The label f_Saccharimonadales indicates that the bacterial taxon was assigned to the order Saccharimonadales; however, classification at the family level was unresolved. In such cases, the order name is provisionally displayed at the family level for taxonomic representation.

These findings indicate that significant differences in multiple bacterial taxa were detected between the groups, clearly demonstrating distinct microbiota structures and substantial changes in the overall microbial composition.

We performed post hoc power analyses for all taxa examined in the study. Most taxa, such as Rhizobiales, *Staphylococcus*, and *Prevotella*, demonstrated high statistical power (power > 0.90), showing that the observed differences were detected reliably even with the limited sample size. In contrast, when we compared taxa such as Bacteroidetes and *Lactobacillus*, the power values were lower (0.50–0.70), implying that some of the non-significant results may have arisen from insufficient power. Detailed results are provided in Supplementary Table S1.

## 4. Discussion

A previous study that aimed to evaluate the short-term effects of orthognathic surgery on periodontal tissues and the oral microbiota investigated the abundance of 11 periodontal pathogens (*Aggregatibacter actinomycetemcomitans*, *Porphyromonas gingivalis*, *Tannerella forsythensis*, *Treponema denticola*, *Prevotella intermedia*, *Peptostreptococcus micros*, *Fusobacterium nucleatum*, *Campylobacter rectus*, *Eubacterium nodatum*, *Eikenella corrodens*, and *Capnocytophaga species*) at three time points: before surgery, 1 week postoperatively, and 6 weeks postoperatively. The results showed that only *Eikenella corrodens* exhibited a temporary increase after orthognathic surgery, whereas no significant changes were observed in the other species [16]; however, that study had several limitations, including the restricted range of bacterial species evaluated, the absence of comprehensive DNA analysis, the lack of assessment of long-term changes in the microbiota, and the unclear impact of antibiotic therapy or other drug administration.

On the other hand, another study investigating the impact of fixed orthodontic appliances on the oral microbiota in Japanese patients reported the collection and analysis of saliva and plaque samples at three time points: before appliance placement, 6 months after placement, and at appliance removal. The study reported clear changes in the composition of the microbiota over time. In particular, periodontal disease-associated anaerobic bacteria, such as *Prevotella*, *Porphyromonas*, and *Fusobacterium*, increased, whereas commensal bacteria commonly found in the healthy oral cavity, such as *Streptococcus*, *Neisseria*, and *Actinomyces*, tended to decrease. These findings suggest that MBAs may shift the oral environment from a healthy state to one with a higher risk of periodontal disease [11].

Moreover, unlike orthodontic treatment alone, the addition of orthognathic surgery in surgical orthodontic treatment introduces factors such as intraoperative and postoperative antibiotic administration; inflammatory responses, including pain and swelling, owing to surgical invasion; and the impact of intermaxillary fixation on the oral hygiene environment. These procedures can lead to restricted mouth opening and difficulty in maintaining oral hygiene, potentially having a substantial effect on the oral environment. Therefore, in this study, participants were classified into Groups S and C, and longitudinal changes in the oral microbiota were compared between the groups.

Our 16S rRNA gene sequencing results revealed a marked increase in *Pseudomonas* at ST2 in Group S. *Pseudomonas* is an opportunistic pathogen and a causative agent of healthcare-associated infections [19]. Although *Pseudomonas* is usually a minor constituent of the oral microbiota, its significant increase at ST2 may be clinically relevant, possibly reflecting a shift toward an opportunistic environment. Antibiotic administration associated with surgical orthodontics, along with inadequate postoperative oral hygiene and the accumulation of plaque and biofilms, may alter the oral microbiota toward a composition dominated by Gram-negative bacilli, thereby facilitating the colonization of pathogenic bacteria [20].

Furthermore, an increase in Saccharimonadaceae detected via the LEfSe analysis in this study has been reported to be associated with periodontitis [21] and may be related to the inflammatory environment following surgical invasion. It has also been suggested that Rhizobiales are associated with mild dental caries and may similarly contribute to postoperative changes in the microbiota [22,23].

We observed increases in *Pseudomonas* and Saccharimonadaceae, but their clinical significance remains unclear. Previous studies have linked Saccharimonadaceae to periodontal inflammation [24,25], and *Pseudomonas* has been reported in peri-implant and postoperative infections [26,27]. However, we cannot yet determine whether these changes directly relate to clinical outcomes such as caries, periodontitis, or surgical site infections. These alterations may suggest a shift toward dysbiosis, but further investigations are required to clarify whether they function as causal contributors or secondary indicators.

Multiple factors that significantly affect the oral environment were present in Group S, including surgical invasion through orthognathic surgery, intraoperative and postoperative antibiotic administration, and difficulty in maintaining oral hygiene due to intermaxillary fixation, postoperative pain, and limited mouth opening. These factors may have promoted the colonization by Gram-negative bacteria and opportunistic pathogens. In contrast, such surgical factors were absent in Group C, which may explain why changes in the oral microbiota were relatively mild in this group when compared with Group S.

All patients who underwent orthognathic surgery in this study received intravenous amoxicillin (1.0 g) during surgery, followed by postoperative intravenous amoxicillin administration for a minimum of 4 days and a maximum of 9 days (mean 4.4 days), at a mean daily dose of 1.8 ± 0.5 g. Amoxicillin is a broad-spectrum β-lactam antibiotic that inhibits bacterial cell wall peptidoglycan synthesis, and its impact on the oral microbiota should be considered. In general, it is considered that changes to the microbiota caused by antibiotics are reversed within 1 week to 1 month after administration, and the oral microbiota is believed to possess inherent resilience to routine disturbances such as antibiotics, tooth brushing, and changes in temperature or oxygen levels [28,29]. Recent evidence suggests that antibiotic exposure can markedly alter the recovery dynamics of the oral microbiota. In a randomized clinical trial of stage III–IV periodontitis patients, investigators found that adjunctive amoxicillin with metronidazole reduced dysbiotic communities dramatically in the short term (from ~86% at baseline to ~2.5% at 2 months), while the placebo group improved only partially (from ~87% to ~62%) [30]. At 26 months, patients in the antibiotic group still exhibited lower levels of dysbiotic taxa compared with baseline (~49% vs. ~86%), but they did not achieve complete restoration to a normobiotic state, and outcomes varied considerably between individuals, with some relapsing after initial improvement [30]. These findings demonstrate that postoperative amoxicillin can strongly influence microbial communities in the short term, whereas long-term recovery of the oral microbiome remains incomplete and heterogeneous across individuals. Because all surgical orthodontic patients in the present study received postoperative amoxicillin, we consider this antibiotic exposure a major confounding factor that may have shaped the microbial shifts observed after orthognathic surgery.

Caries-associated bacteria, such as *Streptococcus* and *Lactobacillus*, have been reported to increase in abundance following the placement of fixed appliances [31]. In this study, we provided oral hygiene instruction when we considered it necessary; however, we did not assess adherence to these instructions either quantitatively or qualitatively. We also did not measure plaque index, gingival index, or self-reported oral hygiene practices, which constitutes a limitation of this study. Nevertheless, no marked increase in these bacteria was observed, likely because saliva sampling began after appliance placement. In contrast, *Neisseria* decreased over time in both groups, although this change was not statistically significant. This result may reflect the role of *Neisseria* as a bacterium that is abundant during the early stages of biofilm formation and decreases naturally over time [11,32].

These results suggest that surgical orthodontics may promote the colonization of Gram-negative bacteria and opportunistic pathogens, which is an important finding highlighting the risk of postoperative infection.

One limitation of this study was the difficulty in standardizing saliva sampling times for all patients. The salivary microbiota is known to be influenced by circadian rhythms; therefore, variability in sampling times may have affected the microbiota composition [33]. In addition, the MBAs had already been removed in some participants at T3 (11 months to 2 years and 4 months postoperatively), and the presence or absence of appliances may have influenced the composition of the microbiota [34]. Furthermore, anatomical and prosthetic conditions, such as the number of teeth and the presence of prosthetic restorations, were not standardized and may have affected the interpretation of the results. We also did not establish standardized criteria for other potential factors that could influence oral microbiota composition, such as dietary habits, sleep patterns, and oral hygiene practices. As these factors may have affected the outcomes, their lack of control represents a limitation of this study. Future studies should include more detailed longitudinal analyses, including the recovery process of the microbiota after appliance removal and the influences of occlusal status and prosthetic conditions.

In this study, we faced an additional limitation of having a relatively small sample size (*n* = 29), which may have reduced our ability to detect effects of small to moderate size. Although our post hoc power analyses showed high power (>0.90) for many taxa with significant differences, some comparisons, including Bacteroidetes and Lactobacillus, showed lower power values (0.50–0.70). These observations suggest that certain non-significant results may stem from insufficient statistical power rather than a genuine absence of biological differences. To validate these findings and secure broader generalizability, future studies with larger cohorts are necessary.

## 5. Conclusions

In the present study, patients who underwent surgical orthodontic treatments exhibited characteristic changes in the oral microbiota when compared with those who received orthodontic treatment alone. In particular, the findings suggested the potential for colonization by opportunistic pathogens, which would not normally be expected to establish in the oral cavity.

Therefore, at each stage of treatment, especially from the preoperative to postoperative phases, active oral hygiene management by dental professionals and patients may be important. These preliminary findings may provide insights for the prevention of plaque accumulation and reducing colonization by opportunistic pathogens. Additional oral hygiene instruction and professional cleaning interventions timed to coincide with ST2, when the most significant changes in the microbiota were observed, as well as strengthened follow-up schedules during this period, could represent potentially beneficial clinical strategies; however, these implications should be interpreted with caution. Further longitudinal and large-scale studies are needed to validate these observations and to determine their clinical applicability.

## Figures and Tables

**Table 1:** Participant characteristics and types of orthognathic surgery.

			Types of Orthognathic Surgery	
	Number of Participants	Age (year) Mean ± SD ^3^	SSRO ^1^	Le Fort I ^2^ + SSRO ^1^
**All study participants**	29	28.2 (±9.3)	—	—
**Group S**	14	29.3 (±9.8)	8	6
Male	5	32.6 (±11.4)	3	2
Female	9	27.4 (±8.2)	5	4
**Group C**	15	27.1 (±8.7)	—	—
Male	4	32.3 (±11.3)	—	—
Female	11	25.3 (±6.6)	—	—

^1^ SSRO: sagittal split ramus osteotomy; ^2^ Le Fort I: Le Fort I osteotomy; ^3^ SD: standard deviation.

## Data Availability

The 16S rRNA sequencing data used in this study were obtained through the analysis service of GENEWIZ from Azenta Life Sciences. The processed data are available from the corresponding author upon reasonable request.

## Supplementary Materials

The following supporting information can be downloaded at: https://www.mdpi.com/article/10.3390/dj13100458/s1, Table S1: Post-hoc power analysis of taxa explicitly mentioned in the text.

## Abbreviations

The following abbreviations are used in this manuscript:

MBAs	Multi-bracket appliances
SSI	Surgical site infection
SSRO	Sagittal split ramus osteotomy
Le Fort I	Le Fort I osteotomy
PCR	Polymerase chain reaction
ASV	Amplicon sequence variant
SD	Standard deviation
LEfSe	Linear discriminant analysis effect size
LDA	Linear discriminant analysis
