# Supplementary material for: Comparison of Long-Term Oral Bacterial Flora Before and After Orthognathic Surgery in Surgical Orthodontic Treatment
Source: Dent J (Basel). 2025 Oct 8;13(10):458. doi: 10.3390/dj13100458 (PMC12562631; doi:10.3390/dj13100458)
Supplement: Supplementary file 1 [file dentistry-13-00458-s001.zip › dentistry-3856393-supplementary.pdf]

## Supplementary Table S1 Post-hoc power analysis of taxa explicitly mentioned in the text

| Comparison | Taxon | p-value | Test type | Effect size r | Converted effect size | Post-hoc Power |
|---|---|---|---|---|---|---|
| ST0 vs ST2 | Rhizobiales (order) | 0.035 | paired (n=14) | 0.563 | dz=1.364 | 0.997 |
| ST0 vs ST3 | *Staphylococcus* (genus) | 0.016 | paired (n=14) | 0.644 | dz=1.683 | 0.9999 |
| ST0 vs ST3 | Rickettsiales (order) | 0.04 | paired (n=14) | 0.549 | dz=1.313 | 0.998 |
| ST1 vs ST2 | Sphingobacteriaceae (family) | 0.022 | paired (n=14) | 0.608 | dz=1.552 | 0.9996 |
| ST1 vs ST2 | *Sphingobacterium* (genus) | 0.022 | paired (n=14) | 0.608 | dz=1.552 | 0.9996 |
| ST1 vs ST2 | *Rhodococcus* (genus) | 0.035 | paired (n=14) | 0.563 | dz=1.364 | 0.997 |
| ST1 vs ST2 | Nocardiaceae (family) | 0.035 | paired (n=14) | 0.563 | dz=1.364 | 0.997 |
| ST1 vs ST2 | Rhizobiales (order) | 0.035 | paired (n=14) | 0.563 | dz=1.364 | 0.997 |
| ST1 vs ST2 | Micrococcales (order) | 0.035 | paired (n=14) | 0.563 | dz=1.364 | 0.997 |
| ST0 vs ST1 vs ST2 | *Treponema* (genus) | 0.007 | paired (n=14) | 0.738 | dz=2.065 | >0.9999 |
| ST0 vs ST1 vs ST2 | Spirochaetales (order) | 0.007 | paired (n=14) | 0.738 | dz=2.065 | >0.9999 |
| ST0 vs ST1 vs ST2 | Fusobacteriia (class) | 0.046 | paired (n=14) | 0.536 | dz=1.266 | 0.998 |
| ST0 vs ST1 vs ST2 | *Campylobacter* (genus) | 0.017 | paired (n=14) | 0.636 | dz=1.655 | 0.9999 |
| ST0 vs ST1 | *Corynebacterium* | 0.042 | paired | 0.545 | dz=1.303 | 0.998 |

| | | | | | | |
|---|---|---|---|---|---|---|
| vs ST2 | (genus) | | (n=14) | | | |
| ST0 vs ST1 vs ST2 | *Capnocytophaga* (genus) | 0.04 | paired (n=14) | 0.549 | dz=1.313 | 0.998 |
| ST0 vs ST1 vs ST2 | *Eikenella* (genus) | 0.026 | paired (n=14) | 0.595 | dz=1.473 | 0.9997 |
| ST3 vs CT3 | Amnipila (genus) | 0.019 | independent (14 vs 15) | 0.441 | d=0.970 | 0.71 |

Supplementary Table S1 (Additions): Post-hoc power analysis for taxa mentioned in the text

Note: For Bacteroidetes, only "p < 0.05" was available; a conservative p=0.05 was used to estimate a lower-bound effect size and power.

Conversion followed the existing table convention: r derived from Z and converted to d (or dz for paired) using $d = 2r / \sqrt{1 - r^2}$. Power computed for two-tailed $\alpha = 0.05$ using a normal approximation to the noncentral t.

| Comparison | Taxon | p-value | Test type | Effect size r | Converted effect size | Post-hoc Power |
|---|---|---|---|---|---|---|
| ST0 vs ST3 | *Prevotella* (genus) | 0.021 | paired (n=14) | 0.617 | dz=1.567 | 0.9999 |
| ST0 vs ST3 | *Veillonella* (genus) | 0.024 | paired (n=14) | 0.603 | dz=1.513 | 0.9998 |
| ST1 vs CT1 | Bacteroidetes (phylum) | <0.05 (assumed 0.05) | independent (14 vs 15) | 0.364 | d=0.782 | 0.5204 |
| ST2 vs CT2 | Bacteroidetes (phylum) | <0.05 (assumed 0.05) | independent (14 vs 15) | 0.364 | d=0.782 | 0.5204 |
| CT0 vs ST0 | *Lactobacillus* (genus) | 0.035 | independent (15 vs 14) | 0.392 | d=0.851 | 0.5941 |
| CT1 vs ST1 | *Lactobacillus* (genus) | 0.023 | independent (15 vs 14) | 0.422 | d=0.931 | 0.6753 |
